# Glucose-6-phosphate dehydrogenase polymorphisms and susceptibility to mild malaria in Dogon and Fulani, Mali

**DOI:** 10.1186/1475-2875-13-270

**Published:** 2014-07-11

**Authors:** Bakary Maiga, Amagana Dolo, Susana Campino, Nuno Sepulveda, Patrick Corran, Kirk A Rockett, Marita Troye-Blomberg, Ogobara K Doumbo, Taane G Clark

**Affiliations:** 1 Malaria Research and Training Centre, Department of Epidemiology of Parasitic Diseases, Faculty of Medicine, Pharmacy and Odonto – Stomatology, USTTB, BP 1805 Bamako, Mali; 2 Department of Molecular Biosciences, The Wenner-Gren Institute, Stockholm University, Stockholm, Sweden; 3 Wellcome Trust Sanger Institute, Hinxton, Cambs, UK; 4 Faculty of Infectious and Tropical Diseases, London School of Hygiene and Tropical Medicine, Keppel Street, London, UK; 5 Centre of Statistics and Applications of University of Lisbon, Campo Grande 1749-016, Lisbon, Portugal; 6 National Institute for Biological Standards and Control, Potters Bar, Herts, UK; 7 Wellcome Trust Centre for Human Genetics, University of Oxford, Oxford OX3 7BN, UK

**Keywords:** Genetic association study, G6PD deficiency, *Plasmodium falciparum*, Malaria, Fulani, Dogon, Mali

## Abstract

**Background:**

Glucose-6-phosphate dehydrogenase (G6PD) deficiency is associated with protection from severe malaria, and potentially uncomplicated malaria phenotypes. It has been documented that G6PD deficiency in sub-Saharan Africa is due to the 202A/376G G6PD A-allele, and association studies have used genotyping as a convenient technique for epidemiological studies. However, recent studies have shown discrepancies in G6PD202/376 associations with severe malaria. There is evidence to suggest that other G6PD deficiency alleles may be common in some regions of West Africa, and that allelic heterogeneity could explain these discrepancies.

**Methods:**

A cross-sectional epidemiological study of malaria susceptibility was conducted during 2006 and 2007 in the Sahel meso-endemic malaria zone of Mali. The study included Dogon (n = 375) and Fulani (n = 337) sympatric ethnic groups, where the latter group is characterized by lower susceptibility to *Plasmodium falciparum* malaria. Fifty-three G6PD polymorphisms, including 202/376, were genotyped across the 712 samples. Evidence of association of these G6PD polymorphisms and mild malaria was assessed in both ethnic groups using genotypic and haplotypic statistical tests.

**Results:**

It was confirmed that the Fulani are less susceptible to malaria, and the 202A mutation is rare in this group (< 1% versus Dogon 7.9%). The Betica-Selma 968C/376G (~11% enzymatic activity) was more common in Fulani (6.1% *vs* Dogon 0.0%). There are differences in haplotype frequencies between Dogon and Fulani, and association analysis did not reveal strong evidence of protective G6PD genetic effects against uncomplicated malaria in both ethnic groups and gender. However, there was some evidence of increased risk of mild malaria in Dogon with the 202A mutation, attaining borderline statistical significance in females. The rs915942 polymorphism was found to be associated with asymptomatic malaria in Dogon females, and the rs61042368 polymorphism was associated with clinical malaria in Fulani males.

**Conclusions:**

The results highlight the need to consider markers in addition to G6PD202 in studies of deficiency. Further, large genetic epidemiological studies of multi-ethnic groups in West Africa across a spectrum of malaria severity phenotypes are required to establish who receives protection from G6PD deficiency.

## Background

Geographical, epidemiological and *in vitro* evidence suggest the hypothesis that glucose-6-phosphate dehydrogenase (G6PD) deficiency confers protection from malaria disease caused by the *Plasmodium falciparum* parasite [1]. G6PD is a key component in the pentose phosphate pathway used by erythrocytes to handle oxidative damage. Following invasion of host erythrocytes, malaria parasites digest haemoglobin to obtain nutrients for their growth. This process releases toxic products, inducing oxidative stress on the cell. G6PD is encoded by a 16.2 kb gene located on the X chromosome. Approximately 160 genetic variants causing clinical deficiency of G6PD have been characterized, and the geographical distribution of these alleles is closely related to populations’ history of exposure to endemic malaria [2].

Establishing whether malaria patients are G6PD deficient is important, because of the future need to use 8-aminoquinolines (e.g., primaquine (PQ) and its derivatives) for malaria elimination in sub-Saharan Africa. PQ is active against all liver stages of Plasmodium, and also offers activity against *P. falciparum* gametocytes, thereby blocking transmission to mosquitoes. However, PQ has low oral bioavailability and is haemotoxic, and can cause haemolytic anaemia in G6PD-deficient individuals. The deficiency can be quantified using enzymatic activity assays, which may be difficult to interpret, especially for mosaic female heterozygotes [3]. Cytochemical methods have been suggested as an alternative [3], but genotyping has been used as a high throughput approach in epidemiological studies. G6PD A- deficiency is the commonest type in sub-Saharan Africa. The presence of 202A/376G alleles (with ~12% reduced enzymatic activity) has been used for association studies to assess the degree to which female heterozygotes and male hemizygotes are protected against severe malaria. Associations between presence of the G6PD202/376 polymorphism and protection against severe malaria have been inconsistent across large studies, observing protective effects in females [4], in males [5,6], in both [7], or no protection [8,9]. The discrepancies could be explained in part by variation in phenotype definition, choice of controls (village surveys *vs* hospital-based studies), age or immune status of subjects, and study designs (case control *vs* cohort) [8]. The techniques used to identify G6PD-deficient individuals are important, as demonstrated by a recent study observing uncomplicated malaria protection only when characterizing deficiency using enzymatic activity from biochemical assays, but not genotyping of G6PD202 [10]. It has also been recognized that allelic heterogeneity has a role in explaining inconsistencies between phenotype and genotype, with evidence from studies in West Africa [5,8] for A-, as well as in Southeast Asia and Oceania for other deficiency types [2]. In the West African setting, the 202A allele frequency across ethnic groups is frequently substantially lower than deficiency rates, and inclusion of alternative G6PD alleles (Santamaria 542 T/376G - ~2% residual enzymatic activity, Betica-Selma 968C/376G - ~11% activity) [11,12] has captured an association with severe malaria in The Gambia [8].

Establishing the region-specific repertoire of G6PD functional variation is required for genetic epidemiological studies. Here G6PD polymorphisms are characterized in Dogon and Fulani ethnic groups in rural Mali (n = 712), where malaria is meso-endemic and transmission rates inside and outside of villages are identical [13]. The polymorphisms are then associated with a non-severe, mild malaria phenotype. Previous studies in West Africa have reported that the Fulani are less susceptible to malaria, compared to other sympatric groups (including Dogon [13,14]), with lower parasite densities, lower incidence and malaria prevalence [13-16]. A previous study of G6PD in the Dogon (and Malinke) ethnic groups in urban Mali has inferred an A-/202A frequency of 7.5% (11.0%) and 16.6% (14.9%) in severe and uncomplicated malaria cases, respectively, leading to protection against severe disease in hemizygous males but not in heterozygous females [5]. From the postulated evolution of G6PD deficiency, it is likely that the frequency of malaria is lower in patients with deficiency. However, a protective effect against uncomplicated malaria has not been conclusively demonstrated.

Fulani have higher rates of spleen enlargement and higher levels of humoral immune responses to a variety of malaria parasite antigens [13-16]. Malarial antigens are sequestered in the spleen in Fulani, leading to higher rates of spleen enlargement compared to other groups, including Dogon [13]. The antigens in the spleen are in contact with (memory) B and plasma cells, leading to higher antibody production, and lower parasite densities and malaria susceptibility in the Fulani [13]. However, in general, immunoglobulin (IgG) antibody responses against *P. falciparum* antigens can be taken as a measure of malaria exposure, and reduced exposure a measure of decreased risk of phenotypes, such as parasitaemia and uncomplicated malaria [17,18]. It has been found that plasmodial infection can also lead to IgE elevation, and not IgG, in children with cerebral malaria compared with patients with uncomplicated disease [19]. There is some evidence suggesting a connection with G6PD deficiency, with a study in Senegal that cell-mediated immune activity may explain the clinical protection afforded by A- deficiency [20]. Here, across the sympatric Dogon and Fulani ethnic groups (n = 712) in the same transmission setting, IgG antibody levels against the *P. falciparum* vaccine candidate antigens apical membrane antigen 1 (AMA1), merozoite surface protein 1–19 (MSP1), merozoite surface protein 2 (MSP2), and circumsporozoite protein (CSP), plus total IgE levels, were quantified. These data allowed an assessment of potential differences in ethnic stimulation of antibody responses, as well as any underlying G6PD A- deficiency mechanisms or genetic effects.

## Methods

The study was performed in a rural village of Manteourou in the African Sahel, where Dogon and Fulani ethnic groups live together in sympatry, within 0.5 km of each other. Cultural and ethnic differences mean that there are no inter-marriages between these two groups. The study was initiated in 2006, and at the time the population size was estimated from the census to be approximately 5,000 inhabitants with 50% Dogon, 45% Fulani and 5% other ethnicities (Rimaibe, Mossi). Two cross sectional surveys were performed, the first at the end of the transmission or rainy season (October/November 2006; n = 594) and the second during the dry season (March/April 2007; n = 345). The study populations (n = 939) included healthy children and adults each from different families, belonging to both ethnic groups (Dogon 53.8%; Fulani 46.2%). In the current study we use the subset of the population (n = 712, 75.8%; Dogon 52.7%; Fulani 47.3%) with DNA available for genotyping. At each survey, clinical (spleen size/enlargement, axillary temperature, body weight) and parasitological data (malaria parasite densities and species), and blood samples were collected (see [14]). Clinical malaria is defined as the presence of fever (axillary temperature of at least 37.5°C) plus the presence of *P. falciparum* parasites on the thick blood smear, in the absence of any other known illnesses. Volunteers visited the health centre at least every month during the season, or were visited by a healthcare if unable to attend the centre, thereby actively followed up for malaria events. If an individual had a malaria event, then that observation was used in the analysis. Repeated events were not considered. Ethical clearance was obtained through the Institutional Review Board of the Malian School of Medicine Pharmacy and Dentistry at the University of Mali. Treatment for malaria and other illnesses detected during the course of the study was provided to the study population at no cost to participants. Community permission was obtained according to the procedures described by previously [21].

Individual written consent was then obtained for each examination or blood collection from the adult or from the child’s parent or care-givers.

**Figure 1:**
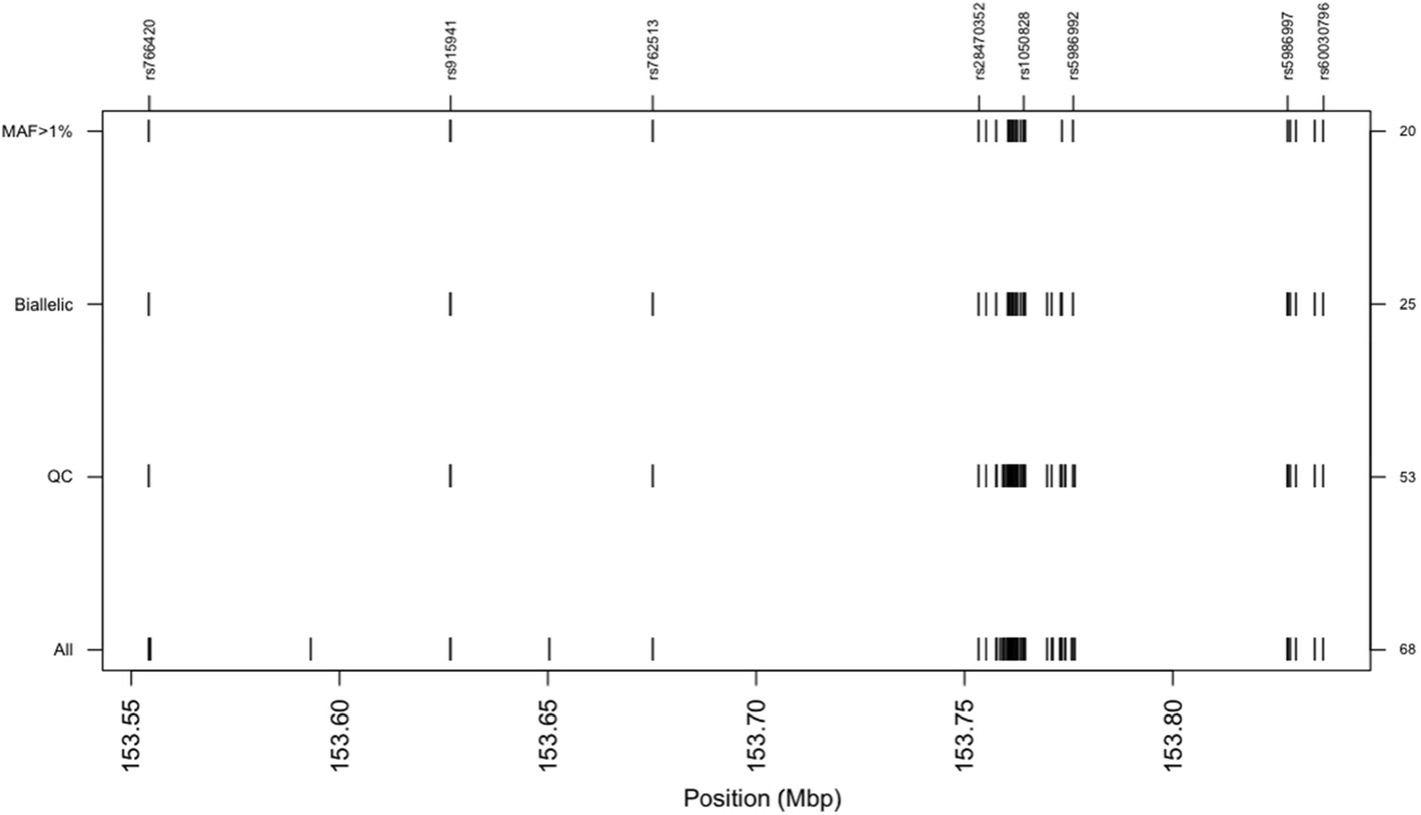
**G6PD map (X chromosome).** MAF = minor allele frequency, right axis has number of SNPs.

All genomic DNA samples (n = 712) were whole-genome amplified by Primer Extension Pre-amplification before genotyping on the Sequenom IPLEX genotyping platform (Sequenom Inc., San Diego, USA) [22], at the Wellcome Trust Centre for Human Genetics, Oxford, as part of an ongoing project (see [14,23] for details). Multiplex design for the iPLEX genotyping methodology was undertaken using the MassARRAY® Assay Design v3.1 Software, and tested using a panel of CEPH and YRI HapMap DNAs [23]. The iPlex genotyping assays included 68 G6PD single nucleotide polymorphism (SNP) positions (described at [23]), HbS (rs334) and HbC (rs33930165), and two SNPs that allow an estimate of the ABO blood group rs8176719, rs8176746). In particular, the rs8176719 derived allele results in a non-functional enzyme, and group O individuals are DD, while non-O Individuals are either II or ID. In addition, rs8176746 is involved in the enzyme’s substrate selection and therefore defines either the A or B blood groups. From blood serum, ELISA was used to detect to total IgE antibody levels [17,18]. ELISA was also used to measure levels of IgG antibodies against *P. falciparum* antigens: AMA1 (FVO, source: Takafumi Tusboi, Ehime University, Japan), MSP1 (K1-Wellcome allele, source: Patrick Corran, LSHTM), MSP2 (DD2, source: David Cavanagh, Institute of Immunology and Infection Research, Edinburgh, UK), and CSP (NANP16 peptide, source: Patrick Corran, LSHTM), (see [18] for a description of assays and data processing). The resulting titre values were log10 transformed to symmetrise them for regression analysis.To assess inter-ethnic group differences for continuous variables (e.g., age in months, immunological titres), Mann–Whitney-Wilcoxon sum-rank tests were applied. Similarly, Pearson’s Chi-square independence tests were applied to categorical variables (e.g., age group, parasite positivity). All analyses involving SNPs were stratified by gender. Genotypic deviations from Hardy-Weinberg equilibrium (HWE) in females were assessed using a Chi-square statistical test. SNPs were excluded from analysis if they had at least 15% of genotype calls missing, more than 2% of males genotype calls were (falsely) called heterozygous, or there was significant deviation from HWE (p < 0.0001). On this basis, 15 SNPs were excluded (rs766420, rs766419, rs743545, rs743548, rs28470352, rs12393550, rs2230037, rs5986990, rs762515, rs73573488, rs2472393, b36_153426256, rs111827785, rs2071429, rs2515904), leaving 53 high-quality SNPs for further analysis (see Figure 1).

The SNP association analysis for the (binary) malaria and clinical phenotypes used a logistic regression model including age group and season as covariates. The association analysis for continuous (log10 transformed) immunological titre phenotypes used linear regression models, and included age group and season as covariates. In all regression models, SNP data were included by fitting a series of genetic models (additive, dominant, recessive, heterozygous advantage, and general), and the minimum p-value reported. The haplotypes of females were inferred from genotypes using an expectation-maximization algorithm [24]. Differences in haplotype frequencies were assessed using Chi-square tests. Haplotype association testing was performed using the regression models described above [24]. Linkage disequilibrium was estimated using the pairwise R-square and D-prime metrics [25]. Differences in linkage disequilibrium (LD) patterns between ethnic groups were assessed using a Bayesian approach [25]. Performing multiple statistical tests leads to inflation in the occurrence of false positives. A Bonferroni correction would be too conservative because all SNPs are from the same gene. A permutation approach that accounted for the correlation between tests estimated that a p-value cut-off of 0.008 would ensure a global significance level of 5%. All association analyses were performed on each ethnic group separately, and where appropriate pooled using a meta-analytical approach. All analyses were performed using the R statistical software, and the *haplo.stat* library was used to implement haplotype analysis.

## Results

The baseline characteristics for the Dogon (n = 375, 52.7%) and Fulani (n = 337, 47.3%) are shown in Table 1. Data from each ethnic group were matched for age, gender and season (all p-values are less than 0.05). There were inter-ethnic differences in frequency of bloods group O (Dogon 44.7%, Fulani 55.6%, known to be protective against malaria [9]) and B (Dogon 30.0%, Fulani 18.0%) (overall P = 0.003). There was also a higher frequency of the HbC-C allele in the Dogon (3.7%) compared to the Fulani (0.6%) (P < 0.001, *Fst* 0.014), but no difference in the HbS–S allele (Dogon 1.5%, Fulani 1.0%, P = 0.615, *Fst* 0.001). Previous work in the predominantly Dogon population of urban Bandiagara has similarly found the frequency of HbC-C is high (8.7%) and that of HbS-S is low (1.6%) [26]. Lower HbC-C frequencies in our study may be due to differences in study settings and designs. As expected, in the Fulani group there were fewer individuals with any clinical malaria case during the season (8.9% *vs* Dogon 16.3%, P = 0.02), less parasite positivity (21.3 *vs* Dogon 28.5, P = 0.03) and hyperparasitaemia (7.4% *vs* Dogon 14.7%, P = 0.003), and greater rates of spleen enlargement (34.4% *vs* Dogon 9.1%, P < 10^-6^). Similarly, all immunoassays showed greater median (geometric mean) levels in the Fulani with all being statistically significant (P < 0.008) in overall analysis, except total AMA1 (P = 0.125). By considering the genotypic surrogates for A- deficiency, inter-ethnic differences were observed in the 202A (Dogon 7.7%, Fulani 0.6%, P < 10^-4^, *Fst* 0.034) and not 376G (Dogon 35.3%, Fulani 35.8%, P = 0.80, *Fst* 0.003) mutations.

**Table 1:** Study characteristics

	**Dogon**		**Fulani**		
	**(n = 375, 52.7%)**		**(n = 337, 47.3%)**		**P-value**
	**N (median)**	**% (range)**	**N (median)**	**% (range)**	
*Baseline characteristics*					
Age in months	(192)	(24 – 744)	(168)	(24 – 900)	0.25
Age groups (years)					0.31
0-4	50	13.3	50	14.8	
5-9	63	16.8	60	17.8	
10-15	70	18.7	77	22.8	
>15	192	51.2	150	44.5	
Male	174	46.4	160	47.5	0.84
Wet Season	298	79.5	259	76.9	0.45
*Genetics*					
Blood Group					0.003*
O	161	44.7	183	55.6	
B	108	30.0	62	18.8	
AB	22	6.1	15	4.6	
A	69	19.2	69	21.0	
HbS					0.62*
AA genotype	358	97.0	327	97.9	
AS genotype	11	3.0	7	2.1	
HbC					< 10^-4^
AA genotype	339	92.4	332	99.1	
AC/CC genotypes	28	7.6	3	0.9	
G6PD202A	29	7.7	2	0.6	< 10^-4^
G6PD376G	130	35.3	109	35.8	0.80
G6PD542A	375	100	337	100	-
G6PD680G	375	100	337	100	-
G6PD968T	375	100	316	93.9	-
*Clinical phenotypes*					
Malaria					0.01*
Clinical	61	16.3	30	8.9	
Asymptomatic	77	20.5	73	21.7	
No malaria	237	63.2	234	69.4	
Hyperparasitaemia	55	14.7	25	7.4	0.003
Parasite positivity	105	28.5	71	21.3	0.03
Spleen enlargement	34	9.1	116	34.4	< 10^-6^
*Immunological pheno.*					
AMA1	(1269)	(0 – 72,770)	(1684)	(3 – 72,770)	0.125
MSP1	(542)	(0 – 131,800)	(2099)	(19 – 356,900)	< 10^-6^
MSP2	(1735)	(0 – 777,500)	(4396)	(49 – 777,500)	< 10^-6^
CSP	(747)	(75 – 779,700)	(1331)	(0 – 1,387,000)	0.0002
Total IgE	(1432)	(0 – 21,780)	(1702)	(171 – 28,960)	0.008

*p-value for an overall effect.

**Figure 2:**
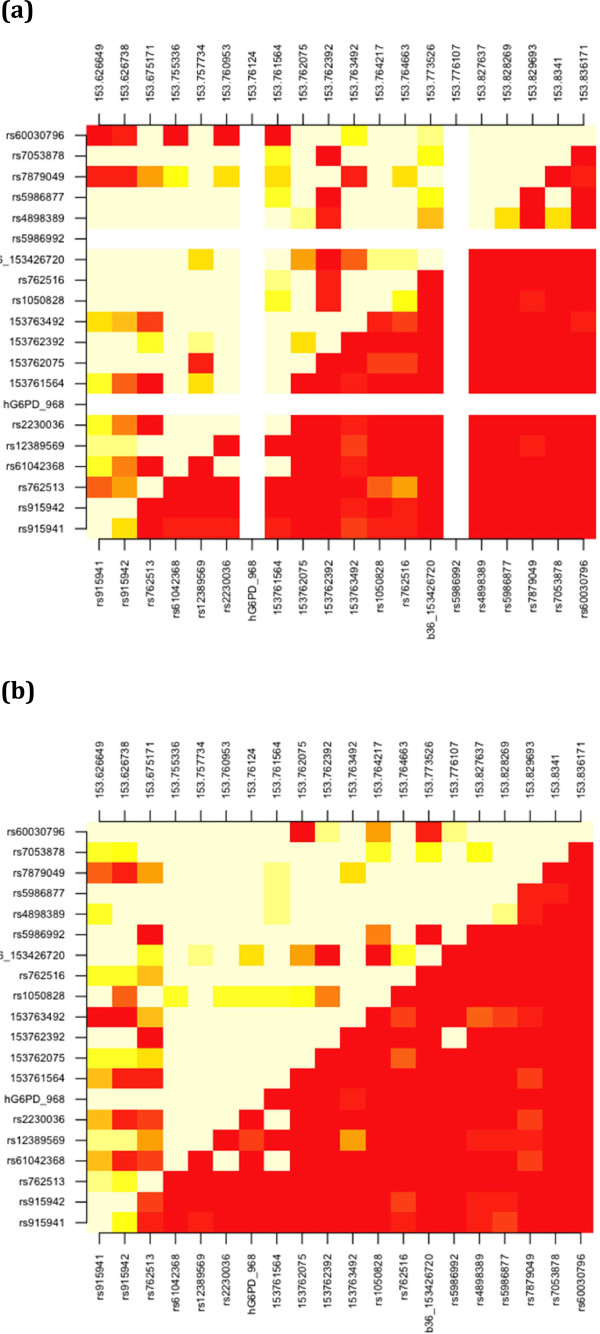
**Pairwise linkage disequilibrium.** (Top left D’, Bottom right R-square; red = 0 - > cream = 1). **(a)** Dogon, **(b)** Fulani. White lines refer to SNPs that are monomorphic.

Of the 53 high-quality G6PD polymorphisms, 28 were found to be monomorphic across ethnic groups (including 680G and rs5030872A/542A, see Tables 1 and 2, Figure 1). Of the remaining 25 SNPs, 12 and 14 had a minor allele frequency in excess of 5% in Dogon and Fulani, respectively. Intra-ethnic gender allele frequencies were similar (results not shown). There were some inter-ethnic differences (*Fst* median 0.015, min. 0.01, max. 0.184), with ten SNPs (including 968 T) having *Fst* values of at least the same magnitude as G6PD202 (0.034). After removing the five SNPs (b36_153413623, rs73641103, b36_153424232, b36_153426720, rs5986997) with minor allele frequencies less than 1%, the remaining 20 SNPs were used to characterize LD across the gene. Pairwise LD was high for Fulani (D’: male median 0.978, range 0.000-1.000; female median 0.981, range 0.003-1.000) and Dogon (D’: male median 0.977, range 0.035-1.000; female median 0.976, range 0.000-1.000) (see Figure 2 for combined gender results). There were LD differences between ethnic groups across the 20 SNPs (Fulani *vs* Dogon P < 0.0001). Based on physical distance, three LD blocks were constructed (Block 1: rs915941, rs915942, rs762513; Block 2: rs61042368, rs12389569, rs2230036, *hG6PD_968*, rs73573478, rs2515905, rs5986875, rs1050829, rs1050828, rs762516, b36_153426720, rs5986992; Block 3: rs4898389, rs5986877, rs7879049, rs7053878, rs60030796). Inter-ethnic differences in haplotype frequencies were observed in each block (P < 0.00001), supporting the observation that Fulani and Dogon have different genetic backgrounds (Table 3) (see Additional file 1 for all haplotype-based results).

**Table 2:** G6PD polymorphisms and minor allele frequencies

**SNP**	**Position**	**Major/Minor allele**	**Dogon**	**Fulani**	***Fst***
			**(n = 375)**	**(n = 337)**	
rs915941	153626649	A/C	0.542	0.352	0.051
rs915942	153626738	G/A	0.435	0.277	0.043
rs762513	153675171	A/G	0.203	0.087	0.040
rs61042368	153755336	G/A	0.152	0.069	0.019
rs12389569	153757734	G/A	0.097	0.209	0.033
b36_153413623**	153760429	G/A	0.011	0.010	0.007
rs2230036	153760953	C/T	0.147	0.068	0.018
**G6PD968**	**153761240**	**T/C**	0.000	0.061	**0.041**
rs73573478	153761564	G/A	0.155	0.074	0.017
rs2515905	153762075	G/A	0.020	0.038	0.004
rs5986875	153762392	G/A	0.009	0.022	0.008
**rs1050829 (376)**	**153763492**	**A/G**	0.353	0.358	**0.003**
**rs1050828 (202)**	**153764217**	**G/A**	0.079	0.006	**0.034**
rs762516	153764663	C/T	0.105	0.112	0.001
rs73641103**	153769889	G/A	0.003	0.000	0.001
b36_153424232**	153771038	T/C	0.004	0.001	0.001
b36_153426354	153773160	A/C	0.027	0.007	0.006
b36_153426720**	153773526	A/G	0.004	0.003	0.004
rs5986992	153776107	C/A	0.000	0.022	0.017
rs5986997**	153827549	C/T	0.003	0.000	0.002
rs4898389	153827637	G/A	0.025	0.312	0.184
rs5986877	153828269	G/C	0.041	0.303	0.154
rs7879049	153829693	A/G	0.512	0.260	0.069
rs7053878	153834100	T/A	0.040	0.064	0.007
rs60030796	153836171	A/G	0.100	0.024	0.034

The following SNPs are monomorphic across both ethnic groups: rs33950507G, b36_153411172C, b36_153412566C, b36_153412620C, b36_153412734G, b36_153412861G, b36_153413455A, rs72554665C, b36_153413799G, b36_153414077G, b36_153414378G, b36_153414531C, b36_153414709C, b36_153414937T, b36_153415014T, **680G**, b36_153415799G, rs5030868G, **rs5030872A (542A)**, b36_153415904C, b36_153416019C, b36_153416656G, b36_153416679A, b36_153417405A, b36_153417417A, b36_153426313G, b36_153427408T, b36_153427466T, b36_153429686G, rs5986997C.

**SNPs with minor allele frequencies less than 1% in each group, and omitted from further analysis.

Bolded are the well known 202, 376, 542, 680 and 968 polymorphisms.

**Table 3:** Haplotype frequencies

Haplotypes****	**Dogon freq.**	**Fulani freq.**
Block 1*		
AGA	0.330	0.640
CAA	0.387	0.201
CAG	0.049	0.076
CGA	0.080	0.073
AGG	0.128	0.006
CGG	0.027	0.004
Block 2**		
GGCTGGGAGCAC	0.484	0.550
GACTGGGGGCAC	0.094	0.148
GGCTGGGGGTAC	0.026	0.070
AGTTAGGAGCAC	0.147	0.065
GAC**C**GGGGGCAC	0.000	0.061
GGCTGAGGGTAC	0.000	0.037
GGCTGGGGGCAC	0.152	0.034
GGCTGGAAGCAA	0.000	0.022
GGCTGAG**GA**TAC	0.019	0.004
GGCTGGG**GA**TAC	0.056	0.002
Block 3***		
GGATA	0.448	0.416
ACATA	0.000	0.239
GGGTA	0.409	0.236
ACAAA	0.025	0.050
GGGTG	0.102	0.023
AGATA	0.000	0.023
GCAAA	0.015	0.013

376G/202A and 968C are highlighted.

*rs915941 rs915942 rs762513.

**rs61042368 rs12389569 rs2230036 hG6PD_968 rs73573478 rs2515905 rs5986875 rs1050829 rs1050828 rs762516 b36_153426720 rs5986992.

***rs4898389 rs5986877 rs7879049 rs7053878 rs60030796.

****Overall differences in haplotype frequencies between ethnic groups within each block are statistically significant (P < 0.000001).

376G/202A and 968C are bolded.

**Figure 3:**
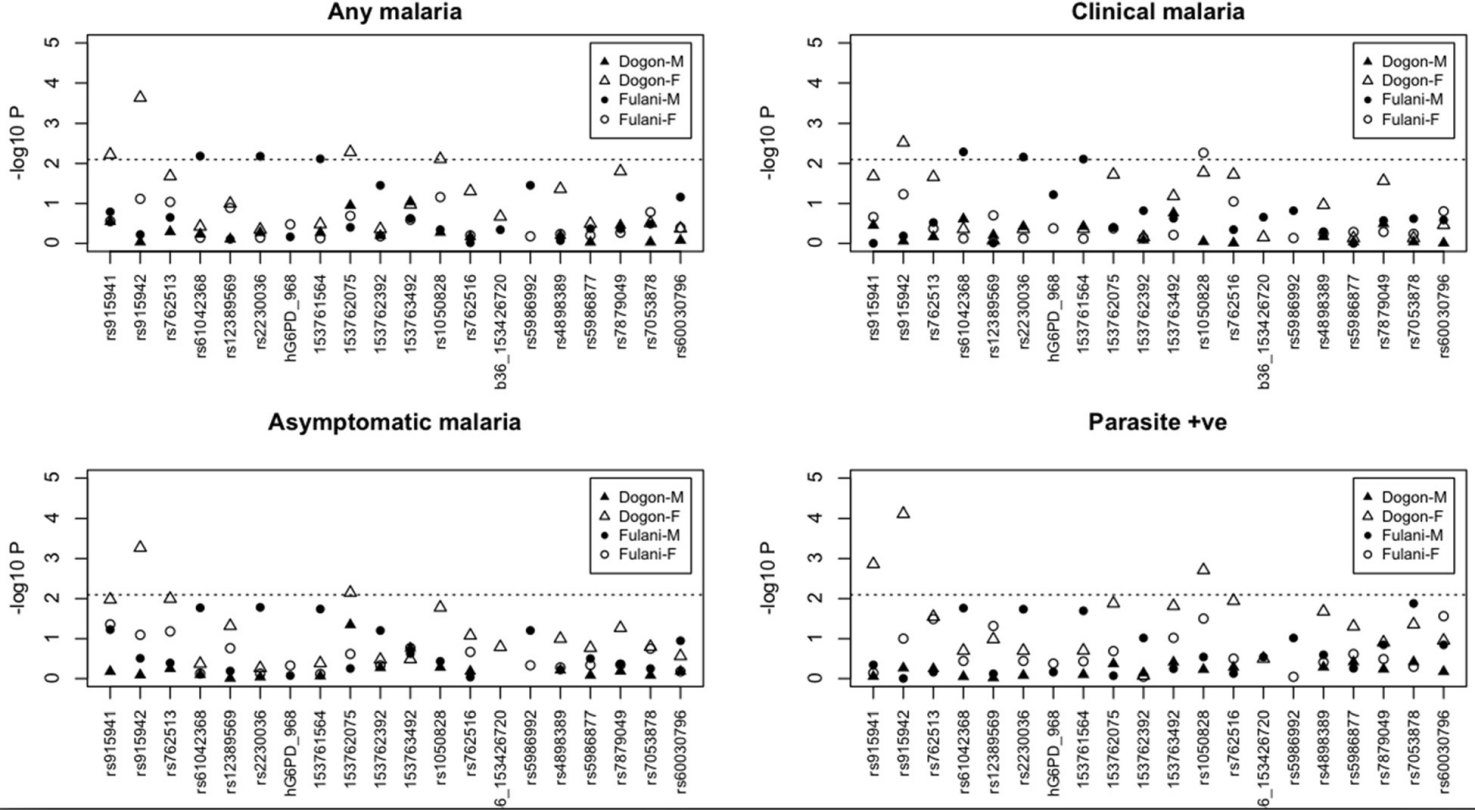
Association analyses for the clinical outcomes.

**Figure 4:**
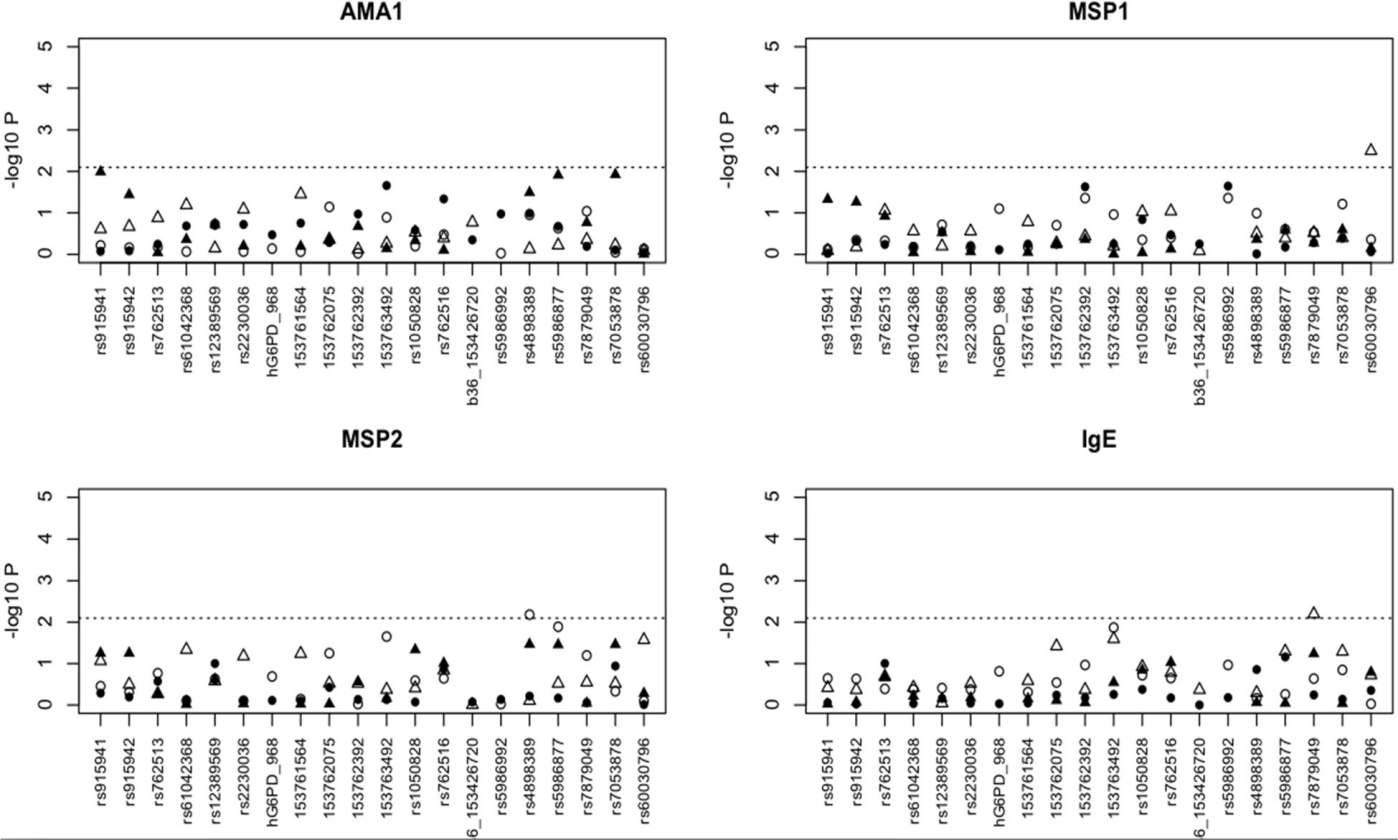
Association analyses for the immunological titres.

SNP-wide association tests were performed for the clinical (Table 4, Figure 3) and immunological phenotypes (Table 5, Figure 4) (see Additional file 2 for all results). For the 202A polymorphism, there were indications of an increased risk of (any non-severe) malaria risk in Dogon (female: OR 3.108 (95% CI 1.295 - 7.460; P = 0.008); male: OR 1.571 (95% CI 0.387 - 6.376; P = 0.528)), but in Fulani the mutation was very rare (< 1%).

**Table 4:** Association analysis* for the clinical phenotypes

				**Association analysis**			
	**Alleles**			**Dogon**		**Fulani**	
**SNP, gender**	**Ref**	**Alt**	**Comparison**	**OR (95% CI)**	**P-value**	**OR (95% CI)**	**P-value**
*Any malaria*							
rs915941, female	C	A	Recessive A	3.193 (1.365, 7.468)	**0.0060**	0.734 (0.418, 1.289)	0.2785
rs915942, female	G	A	Dominance A	0.278 (0.130, 0.595)	**0.0002**	0.163 (0.016, 1.604)	0.0763
rs2515905, female	G	A	Additive A	9.136 (1.262, 66.163)	**0.0052**	0.165 (0.019,1.459)	0.0533
rs1050828, female	G	A	Additive A	3.108 (1.295, 7.460)	**0.0077**	ND	0.0691
rs61042368, male	G	A	A vs. G	0.744 (0.256, 2.159)	0.5843	8.845 (1.474, 53.069)	**0.0065**
rs2230036, male	C	T	T vs. C	0.701 (0.225, 2.185)	0.5376	8.819 (1.469, 52.954)	**0.0066**
rs73573478, male	G	A	A vs. G	0.692 (0.214, 2.233)	0.5347	8.488 (1.413, 50.984)	**0.0077**
*Asymptomatic malaria*							
rs915942, female	G	A	Additive A	0.379 (0.212, 0.676)	**0.0005**	0.544 (0.270, 1.098)	0.0803
rs2515905, female	G	A	Additive A	10.672 (1.447, 78.724)	**0.0071**	0.448 (0.103, 1.947)	0.2412
*Clinical malaria*							
rs915942, female	G	A	Dominance A	0.151 (0.041, 0.554)	**0.0030**	1.924 (0.582, 6.486)	0.0587
rs61042368, male	G	A	A vs. G	0.465 (0.122, 1.766)	0.2451	24.948 (1.951, 319.05)	**0.0051**
rs2230036, male	C	T	T vs. C	0.552 (0.142, 2.151)	0.3805	21.919 (1.713, 280.40)	**0.0069**
rs73573478, male	G	A	A vs. G	0.542 (0.134, 2.185)	0.3775	20.851 (1.637, 265.67)	**0.0078**
*Parasite positivity*							
rs915941, female	C	A	Recessive A	4.512 (1.755, 11.600)	**0.0014**	1.161 (0.507, 2.658)	0.7229
rs915942, female	G	A	Dominance A	0.185 (0.077, 0.445)	**0.0001**	0.178 (0.018, 1.803)	0.0996
rs1050828, female	G	A	Additive A	4.089 (1.626, 10.279)	**0.0019**	ND	0.0315

ND = not determined because of low sample size, *adjusted for age and season.

P-values less than 0.008 are bolded.

**Table 5:** Association analysis* for the immunological phenotypes

**SNP, gender**	**Alleles**			**Association analysis**			
				**Dogon**		**Fulani**	
	**Ref**	**Alt**	**Comparison**	**Slope (95% CI)**	**p-value**	**Slope (95% CI)**	**p-value**
*MSP1*							
rs60030796, female	A	G	Dominance G	0.500 (0.168, 0.832)	**0.0032**	0.286 (-0.440, 1.011)	0.4405
*MSP2*							
rs4898389, female	G	A	Heterozygous	0.094 (-0.581, 0.768)	0.7855	-0.352 (-0.606, -.098)	**0.0066**
*Total IgE*							
rs7879049, female	A	G	Heterozygous	-0.173 (-0.297, -.049)	**0.0064**	-0.044 (-0.169, 0.080)	0.2303

*Adjusted for age and season.

P-values less than 0.008 are bolded.

The potential compensatory effect of the 968C mutation in the Fulani was not statistically significant (male: OR 0.736, 95% CI 0.167-3.245, P = 0.684; female: OR 0.880, 95% CI 0.271- 2.865, P = 0.831). The rs915942 SNP was associated with at least a 60% protective effect against all clinical phenotypes in Dogon females (Table 4, P < 0.005) but not males (P > 0.8) (e.g. any malaria: females OR 0.249, 95% CI 0.116 - 0.537, P = 0.0002; males OR 0.964 95% CI 0.450 - 2.064, P = 0.9244). The rs915941 polymorphism was in high LD with the rs915942, but with a negative correlation, leading to some statistically significant susceptibility effects. Three SNPs in LD (rs61042368, rs2230036, rs73573478) with G6PD968 were associated with increased susceptibility to clinical or any malaria (OR >8) in Fulani males only (females OR >1, p > 0.70) (e.g. for rs61042368 and any malaria: males OR 8.845, 95% CI 1.474 - 53.069, P = 0.0065; females OR 1.220, 95% CI 0.415-3.589, P = 0.7190).

In light of potential associations amongst SNPs, haplotype association tests were performed using the blocks defined above (Additional file 1). Block 1 haplotypes AGG (*vs* CAA OR = 3.751, P < 0.001) and CAG (*vs* CAA OR < 0.100, P < 0.001) were associated with asymptomatic and any malaria in Dogon females, with weaker similar effects in the Fulani (AGG, OR > 10, P < 0.001; CAG OR 0.580, P = 0.387). This result reinforced the finding that haplotypes with the rs915942 mutation are associated with uncomplicated malaria in females. Block 2 contained the rs61042368, G6PD968, 376, and 202 polymorphisms. The haplotype containing the rs61042368 mutation had weak associations with clinical outcomes in Fulani males, but failed to reach statistical significance (P > 0.02). There was some evidence that the haplotype containing 202A (GGCTGAGG[A]TAC) is associated with greater risk of asymptomatic malaria in Dogon (OR 7.189, P = 0.004), but this observation was not statistically significant across all clinical phenotypes, nor was significant for the other related haplotype (GGCTGGGG[A]TAC, P > 0.11). The haplotype containing 968C (GAC[C]GGGGGCAC) in Fulani had non-significant protective effects across all phenotypes, including clinical malaria (*vs* GGCTGGGAGCAC OR 0.673, P = 0.514). For the immunological titres, there were few SNP associations (MSP1 rs60030796; MSP2 rs4898389; Total IgE rs7879049), all in females. Haplotype analysis did not reinforce these effects.

## Discussion

Using 20 high-quality biallelic (of 68) G6PD SNPs, including the established G6PD202, 376, and 968 markers, allele frequencies between the Dogon and Fulani ethnic groups were compared, and tests of association with mild malaria phenotypes performed. There were differences in haplotype frequencies between the ethnic groups, and association analysis did not reveal strong evidence of protective G6PD genetic effects against uncomplicated malaria in both ethnic groups and gender. However, the rs915942 polymorphism (and rs915941 in high LD) was found to be associated with asymptomatic malaria in Dogon females, not males. Similarly, the rs61042368 polymorphism (and rs2230036, rs73573478 in high LD) was associated with clinical outcomes in Fulani males, not females. Whilst one may expect a dose effect that is greater for males than in females, differential protective G6PD polymorphism effects between genders have been observed in studies of severe malaria, and the mechanisms are not fully understood [4-6].

The 202A allele frequencies (7.7%) in Dogon are dissimilar to those reported for controls or uncomplicated groups in urban Bandiagara, Mali (16.6%), but similar to the severe malaria group (7.5%) [5]. These differences could be due to study design (case–control vs. cross-sectional) and setting (urban vs. rural, with no inter-ethnic marriage in the latter). In the present study, whilst the 202A mutation is common in the Dogon, it was found to be rare in the Fulani (~1%). These observations are broadly consistent with a recent small study in the Malian Sahel region of Mopti (Dogon 11.9%, Fulani 2.4%) [27]. In our study, the Betica-Selma 968C/376G, associated with ~11% enzymatic activity, is not present in the Dogon, it has a frequency of 6.1% in Fulani. Similar frequencies of compensatory 968C mutations were found in a mixed Gambian severe malaria case–control study (severe malaria 7.8%, controls 5.4%, overall 6.0%) [8]. In that study, the Fulani comprised 16.1% of participants, and had 202A and 986C mutation frequencies of 3.0 and 6.3%, respectively. A large clinical trial study of over 2,000 individuals across Africa (including Burkina Faso) confirmed the 202A as the most common in Africa, and did not detect 542 T, 680 T or 968C mutations [28]. These findings have been reinforced in a recent study in Burkina Faso (202A 6.0%-14.9%, no 542 T, 680 T or 968C mutations) [29]. However, in both studies it is unlikely that the Fulani are represented in a large number in either study. Preliminary data from a community-based study in Burkina Faso indicates that the Fulani ethnic group has a 202A mutation frequency of ~2% [23], leaving open the possibility of alternative G6PD alleles to explain the prevalence of ~10%. In general, data on the frequency of 542 T, 680 T or 968C mutations is sparse in Africa. Future studies of G6PD deficiency in Africa employing a genetic epidemiological approach may consider using the SNPs characterized here or establish the region-specific repertoire of functional variation using sequencing, before embarking on focused genotyping. This approach is particularly essential in studies comparing G6PD-deficient to -normal patients, for example, when assessing the rates of parasite clearance after treatment with artemisinin-based combination therapy [30].

Differences in malaria phenotypes and immunological titres between Dogon and Fulani ethnic groups have been observed, adding to the growing evidence that the Fulani in West Africa have reduced malaria susceptibility. It is thought that host genetics may play a part, and the role of polymorphism in the G6PD gene was investigated. G6PD A- deficiency is known to associate with reduced risk of severe malaria, and the 202A polymorphism has been used as a genotypable surrogate. Whilst there is strong evidence that G6PD A- deficiency is protective against severe malaria, the effect on mild forms of disease has not been demonstrated conclusively. The results indicate there is no strong evidence of protection to malaria from 202A or 968C mutations in either ethnic group, across gender. This could be explained by the limitations of the sample size, the absence of severe malaria cases in this study, or the lack of sensitivity of genotyping compared to enzymatic assays [10]. However, there was some evidence of increased risk of mild malaria in Dogon with the 202A mutation, but it only attained borderline statistical significance in females. It is possible that this result could be explained by the presence of a flip-flop mutation [31] or allelic heterogeneity [8]. The rs915942 polymorphism (and others in LD) was found to be associated with asymptomatic malaria in Dogon females. The rs61042368 polymorphism (and others in LD) was associated with clinical outcomes in Fulani males, but failed to reach statistical significance in the haplotype analysis. However, the rs61042368 is in high LD with G6PD968, potentially suggesting a similar protective mechanism in mild malaria as seen in severe disease [8,32]. There are no published strong associations between these SNPs and other disease, potentially because in genome-wide studies, polymorphisms in the X-chromosome tend to be somewhat overlooked compared to nuclear polymorphisms. There is sparse data concerning the effect of G6PD polymorphisms on immunological outcomes, with some evidence suggesting that cell-mediated immune activity may explain the clinical protection afforded by A- deficiency [20]. Although there were no associations between 202A, 986C and titre data, three potentially new SNP associations (MSP1 - rs60030796, MSP2 - rs4898389, total IgE - rs7879049) in females were identified. The potential confounding effects of chronic infections on titre values could not be investigated, but like all associations, there is a need for confirmation in follow-up studies.

## Conclusion

Observed genetic differences in the G6PD gene between Fulani and Dogon ethnic groups reinforce the need to consider markers in addition to G6PD202 in studies of deficiency. The SNPs considered in this study provide a starting point for large-scale genetic epidemiological studies of deficiency across Africa. These studies should involve a broad range of ethnic groups, including Fulani, as well as both uncomplicated and severe malaria cases, so that it is possible to establish who receives protection from G6PD deficiency.

## Abbreviations

LD: Linkage disequilibrium; SNP: Single nucleotide polymorphism; G6PD: Glucose-6-phosphate dehydrogenase; PQ: Primaquine.

## Competing interests

The authors declare that they have no competing interests.

## Authors’ contributions

BM, AD; KAR, MT-B, OKD and TGC conceived and designed the study; BM and AD performed recruitment of study subjects; BM, SC, PC, KAR and MalariaGEN coordinated the laboratory experiments; BM, NS and TGC performed the statistical analysis: BM, NS, OKD and TGC wrote the manuscript. The final manuscript was read and approved by all authors.

## Supplementary Material

Additional file 1 All haplotype-based association analyses for the clinical and immunological outcomes, as well as odds ratios for the clinical outcomes. Click here for file

Additional file 2 All SNP-based association analyses for the clinical and immunological outcomes. Click here for file

## References

[B1] Ruwende C Hill A Glucose-6-phosphate dehydrogenase deficiency and malaria J Mol Med 1998 76 581 588 10.1007/s001090050253 9694435

[B2] Tishkoff SA Varkonyi R Cahinhinan N Abbes S Argyropoulos G Destro-Bisol G Drousiotou A Dangerfield B Lefranc G Loiselet J Piro A Stoneking M Tagarelli A Tagarelli G Touma EH Williams SM Clark AG Haplotype diversity and linkage disequilibrium at human G6PD: recent origin of alleles that confer malarial resistance Science 2001 293 455 462 10.1126/science.1061573 11423617

[B3] Shah SS Diakite SA Traore K Diakite M Kwiatkowski DP Rockett KA Wellems TE Fairhurst RM A novel cytofluorometric assay for the detection and quantification of glucose-6-phosphate dehydrogenase deficiency Sci Rep 2012 2 299 22393475 10.1038/srep00299 PMC3293146

[B4] Manjurano AM Clark TG Nadjm B Mtove G Wangai H Sepulveda N Campino SG Maxwell C Olomi R Rockett KR Jeffreys A Riley EM Reyburn H Drakeley C MalariaGen Consortium Candidate human genetic polymorphisms and severe malaria in a Tanzanian population PLoS One 2012 7 e47463 doi:10.1371/journal.pone.0047463. Epub 2012 Oct 29 10.1371/journal.pone.0047463 23144702 PMC3483265

[B5] Guindo A Fairhurst RM Doumbo OK Wellems TE Diallo DA X-linked G6PD deficiency protects hemizygous males but not heterozygous females against severe malaria PLoS Med 2007 4 e66 1 10.1371/journal.pmed.0040066 17355169 PMC1820604

[B6] Santana MS Monteiro WM Siqueira AM Costa MF Sampaio V Lacerda MV Alecrim MG Glucose-6-phosphate dehydrogenase deficient variants are associated with reduced susceptibility to malaria in the Brazilian Amazon Trans R Soc Trop Med Hyg 2013 107 301 306 10.1093/trstmh/trt015 23479361

[B7] Ruwende C Khoo SC Snow RW Yates SN Kwiatkowski D Gupta S Warn P Allsopp CE Gilbert SC Peschu N Natural selection of hemi- and heterozygotes for G6PD deficiency in Africa by resistance to severe malaria Nature 1995 376 246 249 10.1038/376246a0 7617034

[B8] Clark TG Fry AE Auburn S Campino S Diakite M Green A Richardson A Teo YY Small K Wilson J Jallow M Sisay-Joof F Pinder M Sabeti P Kwiatkowski DP Rockett KA Allelic heterogeneity of G6PD deficiency in West Africa and severe malaria susceptibility Eur J Hum Genetics 2009 17 1080 1085 10.1038/ejhg.2009.8 19223928 PMC2986558

[B9] Toure O Konate S Sissoko S Niangaly A Barry A Sall AH Diarra E Poudiougou B Sepulveda N Campino S Rockett KA Clark TG Thera MA Doumbo O Collaboration with The MalariaGEN Consortium Candidate polymorphisms and severe malaria in a Malian population PLoS One 2012 7 e43987 doi:10.1371/journal.pone.0043987. Epub 2012 Sep 5 10.1371/journal.pone.0043987 22957039 PMC3434208

[B10] Johnson MK Clark TD Njama-Meya D Rosenthal PJ Parikh S Impact of the method of G6PD deficiency assessment on genetic association studies of malaria susceptibility PLoS One 2009 4 e7246 10.1371/journal.pone.0007246 19789650 PMC2748715

[B11] De Araujo C Migot-Nabias F Guitard J Pelleau S Vulliamy T Ducrocq R The role of the G6PD AEth376G/968C allele in glucose-6-phosphate dehydrogenase deficiency in the seerer population of Senegal Haematologica 2006 91 262 263 16461316

[B12] Beutler E Kuhl W Vives-Corrons JL Prchal JT Molecular heterogeneity of glucose-6-phosphate dehydrogenase A Blood 1989 74 2550 2555 2572288

[B13] Dolo A Modiano D Maiga B Daou M Dolo G Guindo H Ba M Maiga H Coulibaly D Perlman H Blomberg MT Touré YT Coluzzi M Doumbo O Difference in susceptibility to malaria between two sympatric ethnic groups in Mali Am J Trop Med Hyg 2005 72 243 248 15772314

[B14] Maiga B Dolo A Touré O Dara V Tapily A Campino S Sepulveda N Risley P Silva N Corran P Rockett KA Kwiatkowski D Clark TG Troye-Blomberg M Doumbo OK MalariaGEN Consortium Human candidate polymorphisms involved in malaria susceptibility in sympatric ethnic groups in Mali PLoS One 2013 8 e75675 10.1371/journal.pone.0075675 24098393 PMC3788813

[B15] Modiano D Petrarca V Sirima BS Luoni G Nebie I Diallo DA Esposito F Coluzzi M Different response to *Plasmodium falciparum* in west African sympatric ethnic groups: possible implications for malaria control strategies Parassitologia 1999 41 193 197 10697855

[B16] Modiano D Chiucchiuini A Petrarca V Sirima BS Luoni G Roggero MA Corradin G Coluzzi M Esposito F Interethnic differences in the humoral response to non-repetitive regions of the *Plasmodium falciparum* circumsporozoite protein Am J Trop Med Hyg 1999 61 663 667 10548307 10.4269/ajtmh.1999.61.663

[B17] Corran PH Cook J Lynch C Leendertse H Manjurano A Griffin J Cox J Abeku T Bousema T Ghani AC Drakeley C Riley E Dried blood spots as a source of anti-malarial antibodies for epidemiological studies Malar J 2008 7 195 10.1186/1475-2875-7-195 18826573 PMC2567984

[B18] Proietti C Verra F Bretscher MT Stone W Kanoi BN Balikagala B Egwang TG Corran P Ronca R Arcà B Riley EM Crisanti A Drakeley C Bousema T Influence of infection on malaria-specific antibody dynamics in a cohort exposed to intense malaria transmission in northern Uganda Parasite Immunol 2013 5 164 173 23473542 10.1111/pim.12031

[B19] Perlmann H Helmby H Hagstedt M Carlson J Larsson PH Troye-Blomberg M Perlmann P IgE elevation and IgE anti-malarial antibodies in *Plasmodium falciparum* malaria: association of high IgE levels with cerebral malaria Clin Exp Immunol 1994 97 284 292 8050178 10.1111/j.1365-2249.1994.tb06082.x PMC1534707

[B20] Courtin D Milet J Bertin G Vafa M Sarr JB Watier L Deloron P Troye-Blomberg M Garcia A Migot-Nabias F G6PD a-variant influences the antibody responses to plasmodium falciparum MSP2 Infect Genet Evol 2011 11 1287 1292 10.1016/j.meegid.2011.04.016 21549219

[B21] Diallo DA Doumbo OK Plowe CV Wellems TE Emanuel EJ Hurst SA Community permission for medical research in developing countries Clin Infect Dis 2005 41 255 259 10.1086/430707 15983925

[B22] Ross P Hall L Smirnov I Haff L High level multiplex genotyping by MALDI-TOF mass spectrometry Nat Biotechnol 1998 16 1347 1351 10.1038/4328 9853617

[B23] MalariaGEN Study Website and resources: http://www.malariagen.net

[B24] Lake S Lyon H Silverman E Weiss S Laird N Schaid D Estimation and tests of haplotype-environment interaction when linkage phase is ambiguous Hum Hered 2002 55 56 65 12890927 10.1159/000071811

[B25] Clark TG Campino SG Teo YY Small K Auburn S Rockett KA Kwiatkowski DP Holmes CC A Bayesian approach to assess differences in linkage disequilibrium patterns in genomewide association studies Bioinformatics 2010 26 1999 2003 10.1093/bioinformatics/btq327 20554688 PMC2916719

[B26] Diallo D Traore AK Baby M Rhaly AAG Bellis G Chaventre A Haemoglobinopathies C and S in the dogons Nouv Rev Fr Hematol 1993 35 551 554 8152902

[B27] Dolo A Maiga B Guindo A Diakité SAS Diakite M Tapily A Traoré M Sangaré B Arama C Daou M Doumbo O Frequency of glucose-6-phosphate dehydrogenase deficiency (A-376/202) in three Malian ethnic groups, (in French) Bull Soc Pathol Exot 2014 Epub ahead of print. doi:10.1007/s13149-014-0372-7 10.1007/s13149-014-0372-7 24952161

[B28] Carter N Pamba A Duparc S Waitumbi J Frequency of glucose-6-phosphate dehydrogenase deficiency in malaria patients from six African countries enrolled in two randomized anti-malarial clinical trials Malar J 2011 10 241 doi:10.1186/1475-2875-10-241 10.1186/1475-2875-10-241 21849081 PMC3188486

[B29] Ouattara AK Bisseye C Bazie BV Diarra B Compaore TR Florencia D Glucose-6-phosphate dehydrogenase (G6PD) deficiency is associated with asymptomatic malaria in a rural community in Burkina Faso Asian Pac J Trop Biomed 2014 4 655 658 25183336 10.12980/APJTB.4.2014APJTB-2014-0100 PMC4037660

[B30] Kone AK Sagara I Thera MA Dicko A Guindo A Diakite S Kurantsin-Mills J Djimde A Walcourt A Doumbo O *Plasmodium falciparum* clearance with artemisinin-based combination therapy (ACT) in patients with glucose-6-phosphate dehydrogenase deficiency in Mali Malar J 2010 9 332 doi:10.1186/1475-2875-9-332 10.1186/1475-2875-9-332 21092137 PMC3000419

[B31] Lin P-I Vance JM Pericak-Vance MA Martin ER No gene is an island: the flip-flop phenomenon Am J Hum Genet 2007 80 531 538 10.1086/512133 17273975 PMC1821115

[B32] Sirugo G Predazzi IM Bartlett J Tacconelli A Walther M Williams SM G6PD A- deficiency and severe malaria in the Gambia: heterozygote advantage and possible homozygote disadvantage Am J Trop Med Hyg 2014 90 856 859 doi:10.4269/ajtmh.13-0622 10.4269/ajtmh.13-0622 24615128 PMC4015578

